# Contributions of Experimental Psychiatry to Research on the Psychosis Prodrome

**DOI:** 10.3389/fpsyt.2013.00170

**Published:** 2013-12-17

**Authors:** Mitja Bodatsch, Joachim Klosterkötter, Jörg Daumann

**Affiliations:** ^1^Department of Psychiatry and Psychotherapy, University of Cologne, Cologne, Germany

**Keywords:** experimental psychiatry, psychosis modeling, PCP/NMDA, mismatch negativity, clinical high-risk, prodrome, schizophrenia

## Abstract

In the recent decades, a paradigmatic change in psychosis research and treatment shifted attention toward the early and particularly the prodromal stages of illness. Despite substantial progress with regard to the neuronal underpinnings of psychosis development, the crucial biological mechanisms leading to manifest illness are yet insufficiently understood. Until today, one significant approach to elucidate the neurobiology of psychosis has been the modeling of psychotic symptoms by psychedelic substances in healthy individuals. These models bear the opportunity to evoke particular neuronal aberrations and the respective psychotic symptoms in a controlled experimental setting. In the present paper, we hypothesize that experimental psychiatry bears unique opportunities in elucidating the biological mechanisms of the prodromal stages of psychosis. Psychosis risk symptoms are attenuated, transient, and often only retrospectively reported. The respective neuronal aberrations are thought being dynamic. The correlation of unstable psychopathology with observed neurofunctional disturbances is thus yet largely unclear. In modeling psychosis, the experimental setting allows not only for evoking particular symptoms, but for the concomitant assessment of psychopathology, neurophysiology, and neuropsychology. Herein, the glutamatergic model will be highlighted exemplarily, with special emphasis on its potential contribution to the elucidation of psychosis development. This model of psychosis appears as candidate for modeling the prodrome by inducing psychotic-like symptoms in healthy individuals. Furthermore, it alters pre-attentive processing like the Mismatch Negativity, an electrophysiological component which has recently been identified as a potential predictive marker of psychosis development. In summary, experimental psychiatry bears the potential to further elucidate the biological mechanisms of the psychosis prodrome. A better understanding of the respective pathophysiology might assist in the identification of predictive markers, and the development of preventive treatments.

## Introduction

Since more than 10 decades, researchers aim at understanding the neurobiological mechanisms of psychosis. Concomitant with the debut of modern nosology in the late nineteenth century, almost all pioneer thinkers of psychiatry provided theoretical models regarding the underlying biological mechanisms of psychosis even though the experimental techniques at the time did not allow for any empirical evidence. The paradigmatic claim that “all mental disorders are brain disorders” dates back to Griesinger’s works (1). Later on, Kraepelin, the ancestor of modern nosology, proposed that “dementia praecox” originates in a misdirected neurodevelopment and insisted on the idea that psychosis is identical to a brain disease (2). Bleuler, who gave birth to the name “schizophrenia,” provided a sophisticated theoretical model of disrupted neural association networks contributing to the schizophrenic *Grundsymptome* (3). In this tradition, one of the last hypotheses can be found in the seminal works of the German psychiatrist Gerd Huber, who proposed an elaborated model to trace back subtle psychopathological changes to neurofunctional disturbances of the limbic system (4, 5). Although not all hypotheses of that kind led to fruitful insights, the increasingly elaborated methods of biological psychiatry partially provided empirical evidence for some models in demonstrating, e.g., reductions in brain volume (6), functional aberrations of association cortices (7), and limbic neuropathology (8), thereby justifying the modeling approach as one primary route to guide empirical and experimental work.

However, the empirical work characterized above necessarily represents a kind of “backwards engineering.” Always starting from phenomenology, biological psychiatry is principally conditioned to a verification/falsification dichotomy regarding the primary hypothesis. Thus, this backwards approach is limited by the demarcations given by psychopathology and phenomenological nosology.

In this regard, experimental psychiatry, understood as the modern continuation of ancient theoretical modeling, provides a completive approach to the elucidation of the biological mechanisms of psychosis. The experimental evocation of a psychotic syndrome by psychedelics allows for forward predictions on neurofunctional, cognitive, and psychopathological changes (9). Following this route, experimental psychiatry allows for tracing forwards the consequences of targeted manipulations of neurochemical pathways and for the subsequent comparison with empirical and phenomenological findings.

The birth of experimental psychiatry dates back to the seminal studies of Luby and colleagues (10) who demonstrated that a “schizophrenomimetic” drug evokes psychotic symptoms resembling schizophrenia in healthy individuals (10). Following this approach, Domino et al. were able to evoke comparable symptoms in healthy persons by applying the “dissociative” phencyclidine (PCP)-derivate ketamine (11). In the sequel, empirical findings on the neurochemical features of *N*-methyl-d-aspartate receptor (NMDAR) antagonists as PCP and ketamine first supported the dominating hypothesis of dopamine hyperfunction in schizophrenia (11). However, further evidence demonstrating that *N*-methyl-d-aspartate (NMDA) antagonists critically interact with various regulatory mechanisms of corticolimbic functions that are relevant to schizophrenia led to the establishment of the glutamate hypothesis (11). This hypothesis implies that NMDA-mediated dysfunctions play a critical role not only in dopaminergic regulation, which has been reconceptualized as the final common pathway to psychosis, but particularly predict impairments in cortical, sensory, and associative brain regions that contribute to cognitive and negative symptom dimensions (9). Today, it is widely accepted that the glutamate/NMDA pathway represents a discrete pathophysiological aspect of schizophrenia (9, 12, 13).

However, a yet widely neglected aspect of experimental psychiatry represents its potential contribution to the understanding of psychosis development and pre-psychotic, i.e., prodromal stages [but see Ref. (14, 15)]. This aspect, however, may be of particular interest since the neurobiological mechanisms at the very first, subclinical beginning of psychosis development are yet insufficiently understood (16). Thereby, experimental psychiatry may significantly contribute to the identification of targets for preventive treatments.

In the present review, we aim to investigate if and how experimental psychiatry may further elucidate the biological mechanisms of the psychosis prodrome. Thereto, we will firstly give a short overview on the crucial psychopathological, neurocognitive, and neurofunctional findings in the prodrome with special emphasis on neural information processing [functional magnetic resonance imaging (fMRI), electrophysiology]. Secondly, we will exemplarily focus on the well established PCP/NMDA model of psychosis and its potential to mimic the psychosis prodrome.

## Methods

We carried out a computer search of the MEDLINE database. No limits were set regarding the publication date. We used the following Medical Subject Heading (MeSH) categories: (1) (PCP OR NMDA) AND (psychosis OR schizophrenia), (2) [prediction OR ultra-high-risk (UHR) OR clinical high-risk OR at-risk mental state (ARMS) OR basic symptoms (BS)] AND [psychosis OR schizophrenia], and (3) [neurocognition OR cognition OR fMRI OR P50 OR N100 OR sensory gating OR mismatch negativity (MMN) OR P300] AND [UHR OR prodrome] AND [psychosis OR schizophrenia]. Studies on PCP/NMDA were restricted to human subjects. With regard to the psychosis prodrome, studies were included if current at-risk criteria (COPER/COGDIS, UHR) were employed in the respective studies.

## The Psychosis Prodrome

### Psychopathology

The prodrome of psychosis is at first a phenomenological concept. It originates in the observation that mental disorders, and particularly schizophrenia, mostly do not appear at a sudden. In general, manifest psychosis represents the severe end of a long-term development in which subtle psychopathological changes appear years before a diagnosis can be validly made (17). Since full-blown psychosis represents a severe disorder with critical long-term consequences, the establishment of prediction and prevention based on prodromal signs of psychosis has become a main goal of clinical research. However, although early clinical signs of psychosis development can validly be traced backwards after an individual has already developed full-blown psychosis, the forward prediction of transition to psychosis is difficult and the respective approaches bear substantial uncertainty regarding their predictions (18, 19). In sum, two predictive approaches are currently implemented.

The BS approach points to subtle, subjectively experienced changes of mental functions that are thought to mark the earliest stages of psychosis development (20, 21). Empirical research has led to two well-defined criteria, pointing either to a collection of highly predictive cognitive and perceptive disturbances (COPER) or to predominantly cognitive disturbances (COGDIS), respectively (22). Subjects qualifying for the COPER criterion developed psychosis in 34.9% within 11 months on average (range 1–37, median 9 months) (23).

The currently most widely used clinical criteria of psychosis prediction point to so called UHR symptoms that are thought to mark the latest stages of psychosis development (24, 25). According to this approach, either attenuated psychotic symptoms or brief, spontaneously remitting psychotic symptoms or a genetic liability in combination with an actual loss of functioning indicate a markedly increased risk for an imminent onset of full-blown psychosis (26). Transition rates in samples identified by the aforementioned criteria amount to 30% on average within the available observation periods (27).

Taken together, the prediction of psychosis development based on clinical criteria inherits significant uncertainty, as mirrored by non-conversion rates of more than 50%, at least within feasible observation periods (27). This observation has led to a paradigm shift in that the aforementioned criteria are thought to identify a “risk-state” probably leading to psychosis rather than a “prodrome” mandatorily leading to manifest psychosis (17).

Although the prospective identification of individuals making the transition to full-blown psychosis thus faces major challenges, it is undoubted that the prodromal development commonly starts from subtle changes in perception and cognition and ends up with attenuated and transient psychotic symptoms, respectively, at the verge of manifest psychosis (28). Furthermore, even though not highly predictive of the further course, negative symptoms appear at very early stages of the prodromal development, thereby even preceding (pre-)psychotic symptoms (29). Besides psychopathology, however, recent research has suggested that the prodrome can also be validly characterized on other domains, i.e., neurocognition and neurofunctioning (28).

### Neurocognitive findings

A huge number of studies demonstrated neurocognitive deficits in individuals at-risk and prodromal subjects, respectively. In particular, studies focusing on working memory, executive functions and verbal fluency/learning were able to provide discriminative statistics for the prospective identification of future converters (30–37). Thereby, investigations employing language based tasks demonstrated that verbal fluency deficits precede psychosis onset up to 30 months (30), and that disturbances of working memory can be found up to 64 months prior to psychosis (31). Executive dysfunctions in the prodrome comprise attention and processing speed which appear as well years before psychosis onset (32, 33, 35, 37).

### Neurofunctional findings

Regarding fMRI investigations, yet two studies compared fMRI correlates of neurocognitive functions in converters (i.e., prodromal subjects) to non-converters. Sabb et al. demonstrated a higher activation of temporal lobes, the frontal operculum, the left precentral gyrus, the caudate, and striatal regions of future converters during the semantic logic condition of a language processing task (38). Allen et al. demonstrated an increased activation in future converters, too, with regard to the left superior frontal gyrus, the middle frontal gyrus, parts of the brainstem, and the left hippocampus in a verbal fluency task (39). Taken together with suggestions of a gradual decline in frontal and striatal activation from the clinical risk state to chronic psychosis (40, 41), particularly regions contributing to language processing seem to be involved in prodromal stages (28). Progressive structural changes during transition to psychosis have been found in the superior temporal gyrus (42).

Regarding electrophysiology, the prodrome seems to be characterized by neuronal disturbances in sensory processing domains. Ziermans and colleagues investigated the Pre-Pulse Inhibition (PPI), a startle response, and suggested a differential deficit in converters vs. non-converters (43, 44). Sensory gating measures (P50/N100) seem to be less relevant to the prodromal development since two out of three studies did not find significant differences between converters and non-converters (45–47). The P3 amplitude, which correlates to memory and attentive processes, has been demonstrated to be exclusively disturbed in future converters by one study (48). Of the published studies evaluating the MMN, a correlate of pre-attentive stimulus discrimination presumably sensitive to the stage of illness (49–54), the majority consistently demonstrated MMN deficits in future converters but not in non-converters (55–60). Bodatsch et al. and later on Perez et al. provided evidence that MMN amplitude deficits predict psychosis onset and allow for an estimation of the remaining time until transition (55, 61). Taken together, correlates of sensory processing and pending higher order functions indicate significant disturbances of neural information processing that may characterize the prodrome (28). Thereby, the MMN might be of particular interest regarding future research (62, 63) and early intervention strategies (64).

## The PCP/NMDA Model of Psychosis

### PCP/NMDA and pharmacology

Phencyclidine is a non-competitive antagonist of the NMDA glutamate receptor (NMDAR) (65). Comparable substances are MK801 and ketamine, respectively (66). The binding of PCP at the receptor is state dependent, thereby limited to the open channel state, and shows stereo-selectivity (67, 68). Other channels that can be blocked by PCP are voltage-dependent sodium and potassium channels as well as, with different binding features, the nicotinic acetylcholine receptor (69–71). Interactions with membrane proteins have been identified with regard to opioid receptors, dopamine, and noradrenaline transporters, respectively (72–74). However, the main action site seems to be the NMDAR, since all other effects are less potent and only of minor importance in the clinically relevant doses of PCP (66).

Since NMDA antagonists have been demonstrated to produce schizophrenia-like symptoms, clinical investigations aimed at evaluating the potential therapeutic benefit of glutamatergic agonists (75). Studies investigating naturally occurring agonists employed glycine, d-serine, and d-alanine, respectively (75). The results demonstrate that the combination of one of these agonists with an antipsychotic leads to significant improvements in positive, negative, and cognitive symptom ratings (76–82). In particular, two studies provided preliminary evidence that glycine might lead to partial symptom remission in subjects clinically at-risk of developing psychosis (83). Furthermore, it has been demonstrated that the glutathione precursor *N*-acetyl-cysteine improves MMN deficits in schizophrenia patients (84).

### PCP/NMDA and psychopathology

Since PCP has first been described as “schizophrenomimetic” in the first publications on that topic (10), subsequent research has been able to quantify the respective positive, negative, and cognitive symptoms by psychopathological rating scales (9, 75). Krystal et al. (85) demonstrated that ketamine produces behavior similar to schizophrenia as assessed with the Brief Psychiatric Rating Scale (BPRS) (85). Moreover, individuals suffering from schizophrenia display increases in positive and negative symptom ratings after administration of NMDA antagonists (85, 86). Taken together, the psychopathological observations suggest that NMDA antagonists affect a brain system that is vulnerable to psychotic experiences (9, 75).

However, positive symptoms provoked by ketamine administration have been demonstrated being less severe as those observed in clinical psychosis (85–87). Moreover, some psychopathological characteristics of clinical psychosis seem to be underrepresented in the PCP/NMDA model since, e.g., hallucinations are relatively rare in experimental psychosis (85, 86). However, perception distortion is a typical symptom after ketamine administration (85, 86). In particular, ketamine affects the intensity and integrity of sensory stimuli (85, 88), and salience (85, 88–90), respectively. In turn, negative and disorganized symptoms as alogia and formal thought disorder, respectively, represent specific psychopathological effects of ketamine (91–93).

### PCP/NMDA and cognition

*N*-methyl-d-aspartate antagonists have been demonstrated to produce a wide range of cognitive deficits. Cognitive functions that can be addressed by ketamine comprise predominantly working memory and executive processing (85, 86, 88, 92–96). Moreover, particular performance deficits after administration of subanesthetic doses of ketamine have been shown for learning/cognitive flexibility and verbal fluency (87), which corresponds to the clinical observation of poverty of speech and circumstantiality after ketamine administration (9). The cognitive deficits produced by NMDA antagonists seem to be rather specifically comparable to schizophrenia, since, e.g., a dissociation between disturbed learning ability but intact ability to retain material once learned can be observed in schizophrenia as well as after administration of NMDA antagonists (9, 75, 97).

Taken together, studies demonstrated particularly the induction of working memory impairments and verbal fluency dysfunction in healthy volunteers. These deficits have been pinpointed to ketamine-induced dysfunctions of frontal and temporo-hippocampal parts of the brain (87).

### PCP/NMDA and neural information processing

Deficits in information processing have been reliably demonstrated across methods in schizophrenia. NMDA antagonists have been demonstrated to induce changes in surrogate markers of neural information processing in terms of behavioral and performance changes (9, 15, 75, 87). Furthermore, disturbances of information processing have directly been observed by neurofunctional measures after NMDA antagonist administration. Schizophrenia-like deficits in MMN generation can be induced by local application of NMDA antagonists as well as by systemic administration in healthy individuals (98–102). In contrast, MMN is not modulated by serotonergic or dopaminergic agonists (103, 104). In turn, other brain potentials, e.g., P300, are significantly affected by administration of other psychotomimetic drugs as psilocybin (105). In normal volunteers, however, reduced MMN amplitudes predict susceptibility to ketamine-induced psychosis (98). In schizophrenia, deficits in pre-attentive tone matching might lead to disturbances of higher order functions as the detection of prosody and auditory emotion recognition (9). In turn, in contrast to the results obtained in animal experiments, NMDA antagonists enhance PPI and startle magnitude (106, 107).

With regard to brain imaging studies, it has been demonstrated that ketamine affects metabolic activity in frontal areas, the cingulum, and the thalamus, respectively (90, 108). Furthermore, ketamine induces an increased dopamine release in the striatum of healthy volunteers (109).

## Discussion

### PCP/NMDA and the prodrome

The PCP/NMDA model of psychosis has been demonstrated to be particularly suited to mimic certain aspects of psychosis (9, 11, 75, 87). These aspects straddle basic neurophysiological aberrations as well as neurocognitive and psychopathological features. The psychopathological symptom patterns observed after NMDA antagonist administration have been shown to be largely comparable to clinical psychosis (85, 86). Cognitive deficits produced by NMDA antagonists can similarly be found in schizophrenia (85, 86, 92, 93, 96). Deficits in neurophysiological measures that have been conceptualized as an endophenotype of psychotic disorders have been specifically evoked by the NMDA antagonist ketamine (98, 99).

Although experimental psychiatry has thus proven its ability to advance the neurobiological understanding of schizophrenia, it still faces many criticism. The neurofunctional and psychopathological changes, respectively, evoked in an experimental setting lack many aspects of clinical psychosis (11). Furthermore, experimental psychiatry falls short of modeling the complex brain network disturbances that underlie schizophrenia (11). The evoked changes are moreover transient, thus not able to mimic long term, reciprocal neurodynamics, and represent in sum only partial aspects of the pathophysiological picture. However, mimicking the vast complexity of psychosis in general or schizophrenia in particular shall certainly not be the goal of experimental psychiatry (11). At the foremost, such a modeling approach would distract any well-defined forward predictions. Instead, experimental psychiatry should aim at providing insights in the effects of particular synaptic functions in psychosis (11).

However, it has yet been almost neglected that particularly these alleged imitations of such models provide some unique opportunities in themselves. Psychosis models promoted our understanding of the crucial pathophysiological features of clinical psychosis. Since the prodrome represents “early psychosis,” it can be assumed that the neurobiological properties of the prodrome are at large the very same as in full-blown psychosis, merely at an earlier stage. However, the phenotype of manifest psychosis results not only from the latest differentiation of certain neurobiological alterations, but represents a complex interplay of accumulating developmental factors and progressive pathophysiological changes. This pathophysiological progression defines the later stages of psychosis and makes them distinct from their clinical and biological precursors (110). For example, the pattern of MMN deficits in later stages of schizophrenia has been demonstrated to be different from the early and particularly prodromal stages (111). From a clinical perspective, neurobiological factors at early stages of illness might inform primarily about disease trajectories and prognosis, whereas factors at later stages might inform foremost about persistent pathophysiological mechanisms (110). Experimental modeling of the pre-psychotic stages might thus assist to understand the crucial neurobiological pathways that turn the spontaneously remitting clinical high-risk state into a prodromal development, although these states are clinically indistinguishable (28, 55). However, although models that explicitly address the prodrome of psychosis are yet to come, the existing well established models of psychosis may already provide opportunities for research in this respect.

In synopsis of the literature, the PCP/NMDA model of psychosis displays some properties that brings it close to the prodromal stages of psychosis (see Table 1 for overview). As in the clinical at-risk state, the produced symptoms might resemble in large parts the full-blown psychotic symptomatology (85, 86), but are, however, transient like at least in some high-risk conditions (brief limited intermittent psychotic symptoms). With regard to psychopathology, it has been demonstrated that the positive symptoms induced by NMDA antagonists are less severe than in clinical psychosis (85, 86). This is reminiscent of the attenuated psychotic symptoms that can be found in the prodrome (26). Moreover, changes in the integrity and intensity of sensory perception as well as aberrant salience, as produced by NMDA antagonists (85, 88, 89), might be analogous to the respective BS (21). Besides that, subanesthetic doses of ketamine have been demonstrated to induce particularly negative and disorganized symptoms (91–93) that have been found to precede positive symptoms in the prodromal development (29). Moreover, deficits in verbal fluency and working memory, respectively, which can be evoked by subanesthetic doses of ketamine as well (85, 86, 92–94), have been demonstrated in individuals at-risk and in prodromal stages by neuropsychological and neurofunctional (fMRI) investigations (30–33, 35–39). In clinical studies, the aforementioned cognitive deficits have been demonstrated being predictive of future transition to psychosis in at-risk samples (30–33, 35–37). Finally, the MMN, which has been demonstrated to be predictive of psychosis development (55–58), is rather specifically affected by PCP/NMDA antagonists (9, 75) and altered MMN amplitudes in healthy individuals predicted the individual’s susceptibility to PCP induced psychotic experiences (98). The latter aspect suggests that vulnerability as well as resilience to psychotic experiences might be further understood by a detailed elucidation of NMDA antagonist actions in sensory domains. A close relationship between EEG measures, structural brain changes, and glutamate neurotransmission in the psychosis prodrome has already been demonstrated (112, 113). Counterintuitively, however, a PPI deficit as observed in schizophrenia can not be evoked by NMDA antagonists (107), which might illustrate the limitations of modeling. Following the implications of the glutamate hypothesis, at least two studies have yet been able to demonstrate beneficial effects of the naturally occurring agonist glycine in the at-risk state (83).

**Table 1:** **Comparison of the PCP/NMDA model and the psychosis prodrome**.

Domain	Properties of the PCP/NMDA model	Features of the psychosis prodrome
Psychopathology	Positive symptoms less severe than in clinical psychosis	Attenuated psychotic symptoms
	Impaired integrity/intensity of sensory perception and aberrant salience	Cognitive and perceptive basic symptoms
	Specific negative and disorganized symptoms	Negative/disorganized symptoms precede positive symptoms
Cognition	Verbal fluency deficits and working memory impairments	Verbal fluency deficits and working memory impairments predict psychosis onset
Information processing	Dysfunctions of frontal and temporo-hippocampal parts of the brain	Deficits in frontal cortex, temporal lobes, and hippocampus associated with psychosis onset
	MMN rather specifically affected	MMN predicts psychosis onset and allows for estimating the time until transition

### Contributions of experimental psychiatry to the understanding of psychosis development

At least with regard to the PCP/NMDA model of psychosis, some features advocate in favor of the models potential to advance the understanding of psychosis development. As clinical psychosis, however, not all aspects of the prodrome might be sufficiently represented in such a model. In particular, it is yet only speculative if the symptoms provoked by NMDA antagonists are comparable to prodromal psychopathology (21, 24). Moreover, since psychosis development might proceed via different psychopathological syndromes (21, 24, 26) and more than one pathophysiological pathway (110), it is an open question if and which of these could be best mimicked by psychedelic substances. Furthermore, although much of the neurocognitive and neurofunctional disturbances observed in the prodrome might be evoked by NMDA antagonists, the representation might be rather incomplete.

However, the potential contribution of experimental psychiatry to the understanding of the psychosis prodrome should not be underestimated. As in schizophrenia, prodrome modeling might allow for strong forward predictions and assist in the identification of crucial pathophysiological mechanism as illustrated by the glutamate hypothesis (9, 75), which has been significantly promoted by the results of PCP/NMDA research. Regarding the at-risk state of psychosis, the application of glutamatergic agonists might be understood as a logical consequence of the implications derived from the PCP/NMDA model. Experimental psychiatry might thus not only advance basic research, but assist in the identification of targeted pharmacological interventions in putative prodromal stages of illness.

## Conclusion

A synopsis of the literature shows that the prodrome of psychosis has been almost neglected by experimental psychiatry and the focus has yet been on manifest psychotic disorders. Since prevention of mental disorders became increasingly relevant in the recent decades, it might be fruitful to further evaluate the potential contribution of experimental psychiatry to this goal. As exemplarily illustrated by the PCP/NMDA model of psychosis, however, many aspects advocate that prodromal stages might be validly mimicked by psychedelic substances. In particular, psychopathological as well as neurocognitive and neurofunctional findings in the prodrome seem to be well represented by the PCP/NMDA model. In this regard, future research should aim at comparing the psychopathological properties of putative prodrome models to the respective clinical observations. Furthermore, neurocognitive and neurofunctional effects, respectively, of psychedelics should be evaluated with regard to those deficits that have been demonstrated being predictive of psychosis development.

In summary, experimental psychiatry bears the potential to further elucidate the biological mechanisms of the psychosis prodrome. A better understanding of the respective pathophysiology might assist in the identification of predictive markers, and the development of preventive treatments.

## Conflict of Interest Statement

The authors declare that the research was conducted in the absence of any commercial or financial relationships that could be construed as a potential conflict of interest.
